# Upregulation of SQLE Contributes to Poor Survival in Head and Neck Squamous Cell Carcinoma

**DOI:** 10.7150/ijbs.68216

**Published:** 2022-05-16

**Authors:** Jing Li, Tao Yang, Qihong Wang, Yuedan Li, Haiyan Wu, Mei Zhang, Hong Qi, Hongxin Zhang, Jinfeng Li

**Affiliations:** 1 Key laboratory of Shaanxi Province for Craniofacial Precision Medicine Research, College of Stomatology, Xi'an Jiaotong University, 98West 5th Road, Xi'an, Shaanxi 710004, China.; 2 Department of Stomatology, Shaanxi Provincial Hospital, Xi'an, Shaanxi, 710038, China.; 3 Department of Pain Treatment, Tangdu Hospital, Fourth Military Medical University, Xi'an, Shaanxi, 710038, China.; 4 Clinical Medical Research Center, The 75 th Group Army Hospital, Dali 671000, China.; 5 Department of Pharmacy, General Hospital of Central Theater Command, Wuhan 430010, China.; 6 Department of Cleft Palate-Craniofacial Surgery, College of Stomatology, Xi'an Jiaotong University, 98West 5th Road, Xi'an, Shaanxi 710004, China; 7 Lianhu clinic, Affiliated Stomatological Hospital of Xi'an Jiaotong University, Xi'an, 710003, China.; 8 Department of Ultrasound Diagnosis, Laoshan branch of NO.971 Hospital of the people's liberation army navy, Qingdao 266100, China.; 9 Department of Pathology, Xi'an Jiaotong University College of Stomatology, 98West 5th Road, Xi'an, Shaanxi 710004, China.; 10 Department of Intervention Therapy, The Second Affiliated Hospital, Shaanxi University of Traditional Chinese Medicine, Xianyang 712046, China.

**Keywords:** SQLE, HNSCC, tumor progression, prognosis, terbinafine

## Abstract

Recently, increasing attention has been paid to the role of Squalene epoxidase (SQLE) in several types of cancers. However, its functional role in tumor progression of head and neck squamous cell carcinoma (HNSCC) is still unclear. We performed bioinformatic analyses and relative experiments to assess the potential mechanism of SQLE-mediated HNSCC malignancy. And the results showed that SQLE was significantly upregulated in tumor samples compared with peritumor samples. Mechanistically, miR-584-5p downregulation may lead to the upregulation of SQLE in HNSCC. Moreover, high SQLE expression in HNSCC was associated with TNM stage, distant metastasis, and poor survival, indicating that SQLE be involved in the progression of HNSCC. Furtherly, SQLE boosted proliferation, migration, invasion of HNSCC cells *in vitro* and* in vivo*. Bioinformatic studies showed that PI3K/Akt signaling participated in HNSCC progression mediated by SQLE overexpression, which is confirmed by *in vitro* and* in vivo* analysis. Particularly, treatment with terbinafine, an inhibitor of SQLE widely used in the treatment of fungal infections, showed a therapeutic influence on HNSCC. Our findings demonstrate that SQLE plays a vital role in HNSCC progression, providing research evidence for SQLE as a prospective HNSCC therapeutic target and for terbinafine as a candidate drug of HNSCC treatment in the future

## Introduction

Head and neck squamous cell carcinoma (HNSCC) accounts for more than 5% of all malignancies 1. Over the past two decades unfortunately, overall disease outcomes have not been significantly improved despite considerable advances in the surgical and medical treatments. This is due to many newly diagnosed HNSCC patients with advanced stage disease at diagnosis. More importantly, approximately 60% of patients develop with locoregional recurrence and 15-25% of patients with distant metastasis, which contributes to most treatment failure in cases of HNSCC 2, 3. Thus, there is still an urgent need to identify new key molecules which are involved in the progression of HNSCC.

Recently, tumor biological function of several metabolic key enzymes has attracted interest. The gene *SQLE* encodes squalene epoxidase, which is one of the key enzymes and catalyzes the oxidation of squalene to 2,3-oxidosqualene. It is well known that cholesterol is critical to the growth and migration of tumor cells and maintaining optimal levels of cholesterol is requisite for cellular function and viability 4. Several studies have illustrated that SQLE could promote tumor progression via synthesis of cholesterol 5, 6. Also, Liu et al. preliminarily reported SQLE upregulation in HNSCC cells might predict poor prognosis 7. However, as far as we know, the functional mechanisms of SQLE in HNSCC has not been reported.

In the present study, we systematically investigated the expression, clinical roles and functional mechanisms of SQLE in HNSCC, with hopes of providing useful dimension into the progression of HNSCC. This study provides new insights to realize the pathological roles of SQLE in HNSCC progression and provides preliminary evidence for SQLE as a potential therapeutic target in HNSCC.

## Materials and Methods

### Data resource

GEO microarray series (GSE33232 8, GSE59102 (no publication), GSE107591 9, GSE136037 10) were obtained from NCBI's database of gene expression (GEO, https://www.ncbi.nlm.nih.gov/geo/). Platforms and samples of GEO series were listed in **Table 1**.

### Bioinformatics analysis for identifying SQLE expression

Studies comparing SQLE between tumor and non-tumor samples in HNSCC were selected with a threshold of *p*-value ≤ 1e-4, fold change ≥ 2 and top 10% gene rank in the Oncomine database (https://www.oncomine.org/)^11^.Raw microarray data of each GEO dataset were normalized by Robust Multichip Analysis (RMA) from the R affy package 12. SQLE expression was compared between tumor and non-tumor samples from the R Limma package 13. HNSCC RNA-Seq data from TCGA were analyzed through UALCAN website (http://ualcan.path.uab.edu/)^14^, which was comprised of 520 tumor samples and 44 non-tumor samples.

### Survival analysis

The clinical information and SQLE RNA expression data of 568 TCGA-HNSCC patients were downloaded from the cBioPortal database 15 (http://www.cBioPortal.org/). After deleting some missing data, the SQLE expression values were ranked in descending order and the median was taken as the cutoff value, and then patients were assigned into the low and high expression groups to evaluate the correlation between SQLE expression and survival rates.

### Targeted microRNA analysis

MicroRNAs targeted SQLE were predicted by miRWalk (http://mirwalk.umm.uni-heidelberg.de/) 16, microRNA Data Integration Portal (mirDIP) (http://ophid.utoronto.ca/mirDIP/) 17 and Targetscan (http://www.targetscan.org/vert_71/) 18 database.

### Identification of SQLE-related genes

RNA-Seq data information from TCGA-HNSCC patients was divided due to the correlation with SQLE expression into two groups to screen for SQLE-related genes. Those genes chosen by the cut-off criteria of* p*-value<0.01 and |logFC|>2 were defined as SQLE-related genes.

### Biological process enrichment

The Database for Annotation, Visualization, and Integrated Discovery (DAVID, http://david.ncifcrf.gov) 19 was applied for pathway enrichment analysis. Top 10 annotation terms were shown.

STRING (http://string-db.org) 20 was used to analyze the interactions between SQLE and those related proteins. The minimum required interaction score was defined as 0.4 and the protein nodes which underwent no interaction with other proteins were removed.

### Cell culture and tissue collection

Keratinocytes (HaCaT) and HNSCC cells (FaDu and CAL-27) were from the National Infrastructure of Cell Line Resource (Beijing, China). HSC3 cells were from the Health Science Research Resource Bank (HSRRB, Japan Health Sciences Foundation (Osaka, Japan). HNSCC cells (TSCCa, SCC9, SCC25) were purchased from the Procell Life Science &Technology Co.,Ltd. (Wuhan, China). A total of 115 paired HNSCC tissues were collected at Xi'an Jiaotong University College of Stomatology in the present study, which was approved by the Ethics Committee of Xi'an Jiaotong University College of Stomatology. Moreover, all participants enrolled in the study have signed informed consent. For the clinical data, please see** Table 2**.

### Quantitative Real-Time PCR

Total RNA was extracted from tissues and cell lines using RNAiso Plus kit (Takara, Tokyo, Japan). The RNA sample was reverse transcribed into cDNA using PrimeScript™ RT Master Mix kit (Takara, Tokyo, Japan). SQLE, β-actin, miR-584-5p, U6 primers were synthesized by Sangon Biotech (Shanghai) Co., Ltd. The quantitative real-time PCR (qRT-PCR) experiment was conducted using SYBR Premix Ex Taq Kit (Takara, Tokyo, Japan) with a Bio-Rad Real-time PCR system. The data were analyzed using the relative 2^-ΔΔCT^ method. The primers used are listed as below. SQLE (Forward (5' to 3'): TGACAATTCTCATCTGAGGTCCA; Reverse (5' to 3'): CAGGGATACCCTTTAGCAGTTTT); β-actin (Forward (5' to 3'): GACCTGACTGACTACCTCATGAAGAT; Reverse (5' to 3'): GTCACACTTCATGATGGAGTTGAAGGT); miR-584-5p (Forward (5' to 3'): TTATGGTTTGCCTGGGACTGAG; Reverse (5' to 3'): miRscript universal primer), miR-1183 (Forward (5' to 3'): ACTGACCACTGTAGGTGATGGT; Reverse (5' to 3'): GCGAGCACAGAATTAATACGACTCACTATAGG), miR-3127-3p (Forward (5' to 3'): ATTCCCCTTCTGCAGGCC; Reverse (5' to 3'): GCAGGGTCCGAGGTATTC), miR-1298-5p (Forward (5' to 3'): ACACTCCAGCTGGGTTCATTCGGCTGTCCA; Reverse (5' to 3'): TGGTGTCGTGGAGTCG), U6 (Forward (5' to 3'): CTCGCTTCGGCAGCACA; Reverse (5' to 3'): AACGCTTCACGAATTTGCGT).

### Western blot and immunohistochemistry

***Western blot (WB) analysis****.* Cells or tissues were lysed in cold RIPA buffer. Approximately 60 μg total protein were loaded per lane and electrophoresed by SDS-PAGE, and then transferred onto PVDF membrane, which was blocked in 5% non‑fat milk and incubated with the corresponding antibodies. Finally, the protein bands were detected using an enhanced chemiluminescence system (Clinx Science Instruments Co., Ltd., Shanghai, China). Primary antibodies used are listed: β-actin (#66009-1, dilution: 1/3000, Proteintech); SQLE (#12544-1-AP, dilution: 1/1000, Proteintech); CDK4 (#11026-1-AP, dilution: 1/500, Proteintech); CDK6 (#14052-1-AP, dilution: 1/1000, Proteintech); Cyclin D1 (#60186-1-AP, dilution: 1/1000, Proteintech); cleaved caspase-3 (#ab2302, dilution: 1/500, Abcam); cleaved caspase-9 (#ab2324, dilution: 1/1000, Abcam); cleaved PARP (#ab194217, dilution: 1/1000, Abcam); E-cadherin (#20874-1-AP, dilution: 1/500, Proteintech); N-cadherin (#13116, dilution: 1/1000, Cell Signaling); ZO-1 (#21773-1-AP, dilution: 1/500, Proteintech); Vimentin (#10366-1-AP, dilution: 1/1000, Proteintech); PI3K(#20584-1-AP, dilution: 1/500, Proteintech); p-PI3K(#17366, dilution: 1/1000, Cell Signaling); Akt(#10176-2-AP, dilution: 1/1000, Proteintech); p-Akt(#66444-1-Ig, 1/1000, Proteintech); MSMO1(#ABIN310847, dilution: 1/1000).

***Immunohistochemistry (IHC) analysis.*** IHC analysis was implemented on 4‑μm paraffin‑embedded HNSCC sections using an MaxvisionTM2 HRP-Polymer anti-Mouse/Rabbit IHC Kit (MXB Biotechnologies, Fuzhou, China). Primary antibody against SQLE (#12544-1-AP, dilution: 1/200) was purchased from Proteintech Group, Inc. The target protein expression levels were blindly assessed according to the proportion and intensity of positive cells.

### Knockdown and forced expression of SQLE

The following siRNAs were from GenPharma (China). siRNA sequences are listed below: Control siRNA: 5'-UUCUCCGAACGUGUCACGUTT-3'; SQLE#1: 5'-GCCUCUAAAUCUUUAGGUUTT-3'; SQLE siRNA#2: 5'-GCCCAGGUUGUAAAUGGUUTT-3'. siRNAs were transfected into cells using Lipo3000 transfection reagent (Invitrogen, United States).

Overexpression plasmids for SQLE were generated by cloning the ORF into the pcDNA3.1(+) vector and applied to transfect into cells using Lipo3000 transfection reagent (Invitrogen, United States). Two days after the transfection, the transfected cells were cultured in the presence of 500μg/ml Neomycin (Invitrogen, United States) for 10 days. Finally, stable colonies were collected. The knockdown efficiency and specificity of siRNAs, ASO and shRNAs were validated by either RT-qPCR or immunoblots.

### Dual-Luciferase Reporter Assay

The dual-luciferase reporter assay was performed as previously described 21. Briefly, the wild and mutant SQLE 3'-UTR were cloned into the reporter plasmid. HNSCC cells were co-transfected with plasmids and miR-584-5p mimics or negative control using Lipo3000 (Invitrogen, United States). After 48 h, activity of the Firefly and Renilla luciferase was assessed. The relative luciferase activity was normalized to Renilla fluorescence.

### In vitro assays for cell proliferation

Cell proliferation was determined by MTS assay. Briefly, HNSCC cells were plated in 96-well plates (Costar) at 1000 cells per well. After 12 h, cell viability was measured by MTS addition and incubation for 2 h at 37 °C. The microplates were read at 490 nm wavelength. Each sample was analyzed in triplicate. For terbinafine treatment, 5000 HNSCC cells were plated in 96-well plates per well and treated with terbinafine in the range of 200 μM-100 nM. After 72 h, viability was determined by MTS assay.

### In vitro assays for cell migration and invasion

Cell migration and invasion were assessed by wound healing and transwell matrigel invasion assays, respectively. In brief, for would healing assay, scratch wounds were conducted using plastic pipette tips, and the wound healing was observed at 0 and 48 h. For invasion assay, 1×10^5^ of HNSCC cells were plated in the upper chamber and cultured with medium containing 10 % fetal bovine serum in the bottom chamber for 48 h. Invaded cells were then stained with crystal violet.

### Cholesterol content measurement

HNSCC cells homogenates were prepared for cholesterol extraction using ethanol and then quantitatively measured for the levels of cholesterol with a commercial kit from Jiancheng (A111-1-1, Jiancheng, Nanjing, China), following their manufacturers' protocols.

### In vivo assays for murine HNSCC tumorigenesis, tumor growth and metastasis analyses

All animal experimental procedures were approved by the Ethics Committee of Xi'an Jiaotong University College of Stomatology. Male 5-6-week-old C57BL/6J mice were given drinking water containing 4-nitroquino⁃line-l-oxide (4-NQO) (200 g/mL) and arecoline (500 g/mL) for 16-20 weeks. Primary HNSCC tumors were harvested and lymph nodule metastases were collected using 0.1 mL methylene blue injection and histological examination as conformations. Then the corresponding HE and IHC assays were conducted. Male 5-6-week-old Balb/c nude mice with body weight of 18-22 g were raised under standard conditions and randomly divided into groups (6 mice/group). For tumor growth assay, 1×10^7^ CAL-27-shSQLE or SCC9-SQLE cells were injected subcutaneously into right flank of the Balb/c nude mice (6 mice/group). Tumor volume (mm^3^) was assessed every 5 days in a 6-week period. The mice were sacrificed after which the tumor nodules were harvested and photographed. Ki67 and TUNEL staining assays were used to evaluate proliferative and apoptotic phenotypes. For *in vivo* metastasis assay, 1×10^6^ cells were injected into the tail vein of nude mice. After 6 weeks, the mice were sacrificed and lungs were dissected and prepared for standard histological examination.

### Statistical analysis

Experiments were repeated 3 times, where appropriate. SPSS 17.0 software (SPSS, Chicago, IL) was used for all statistical analyses and *p*<0.05 was considered significant. Unpaired t tests were used for comparisons between 2 groups where appropriate.

## Results

### SQLE is upregulated in HNSCC tissues via bioinformatic analysis

**Figure 1:**
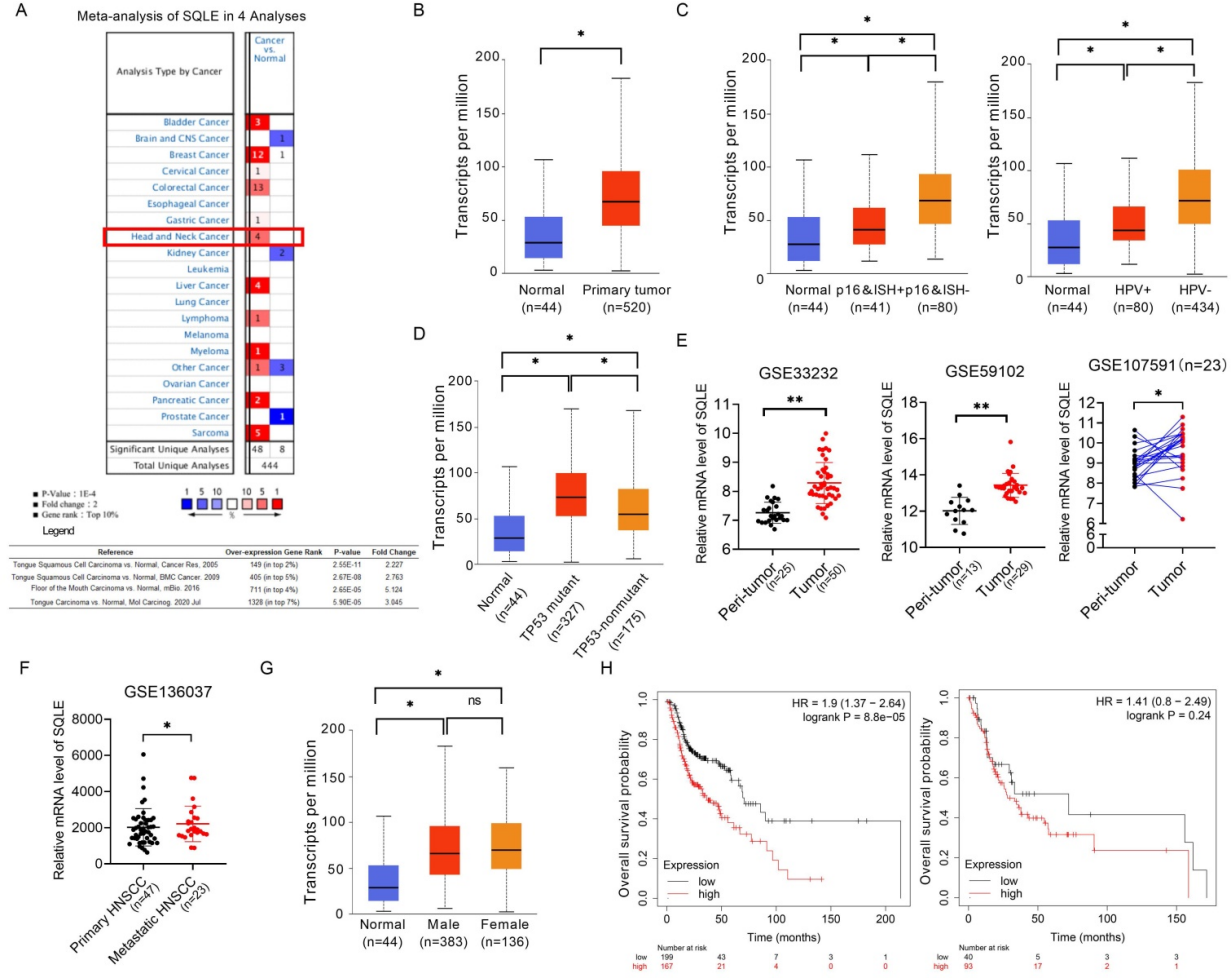
** SQLE mRNA level is commonly up-regulated in HNSCC tissues based on bioinformatic analysis.** (A) Meta-analysis of SQLE expression based on Oncomine database. (B) mRNA expression of SQLE from TCGA-HNSCC data. (C) SQLE mRNA expression analysis based on HPV infection subgroup from TCGA-HNSCC data (Left panel: p16&ISH; Right panel: HPV virus infection). (D) SQLE mRNA expression analysis based on TP53 mutation subgroup from TCGA-HNSCC data. (E) Expression of SQLE in GSE33232, GSE59102 and GSE107591 datasets. (F) Expression of SQLE in GSE136037 dataset. (G) SQLE mRNA expression analysis based on the factor of genders from TCGA-HNSCC data. (H) Kaplan-Meier assessment for overall survival (OS) according to genders in TCGA-HNSCC dataset. Abbreviation: p16&ISH, p16 immunohistochemistry and HPV in situ hybridization; HNSCC, head and neck squamous cell carcinoma.

We compared the mRNA level of SQLE in cancers with that in normal samples using Oncomine databases. SQLE mRNA level was significantly upregulated in patients with HNSCC in four datasets, with a fold change of 2.227 to 5.124 (Figure 1A). Using the UALCAN database (http://ualcan.path.uab.edu/), we compared SQLE expression between HNSCC and peri-tumor tissues. The results indicated that SQLE expression was higher in HNSCC tissues than in normal tissues (Figure 1B). Together from the UALCAN dataset, we also analyzed expression difference of subgroups according to HPV infection and TP53 mutant. On the one hand, we found SQLE expression was higher in HPV-negative HNSCC tissues based on detection by p16 immunohistochemistry and HPV in situ hybridization (ISH) (p16&ISH) and by PCR reaction (Figure 1C). On the other hand, we found SQLE expression is higher in TP53-mutant group compared to TP53-nonmutant group (Figure 1D). Furtherly, we found that SQLE expression was upregulated in HNSCC patients in two non-paired GEO dataset (GSE33232 and GSE59102) and a paired GEO dataset (GSE107591) (Figure 1E). Interestingly, we also investigated SQLE expression in GSE136037 dataset containing primary and metastatic HNSCC tissues and found SQLE expression is upregulated in metastatic lesions, indicating the promoting roles of SQLE in both tumor growth and metastasis (Figure 1F). Also, we found male patients (HR=1.9 (1.37-2.64), log rank *P*=8.8e-05) were supposed to have poorer OS compared to female patients (HR=1.41(0.8-2.49), log rank *P*=0.24) (Figures 1G-H).

### SQLE is upregulated in HNSCC tissues and contributes to tumor progression and worse prognosis

**Figure 2:**
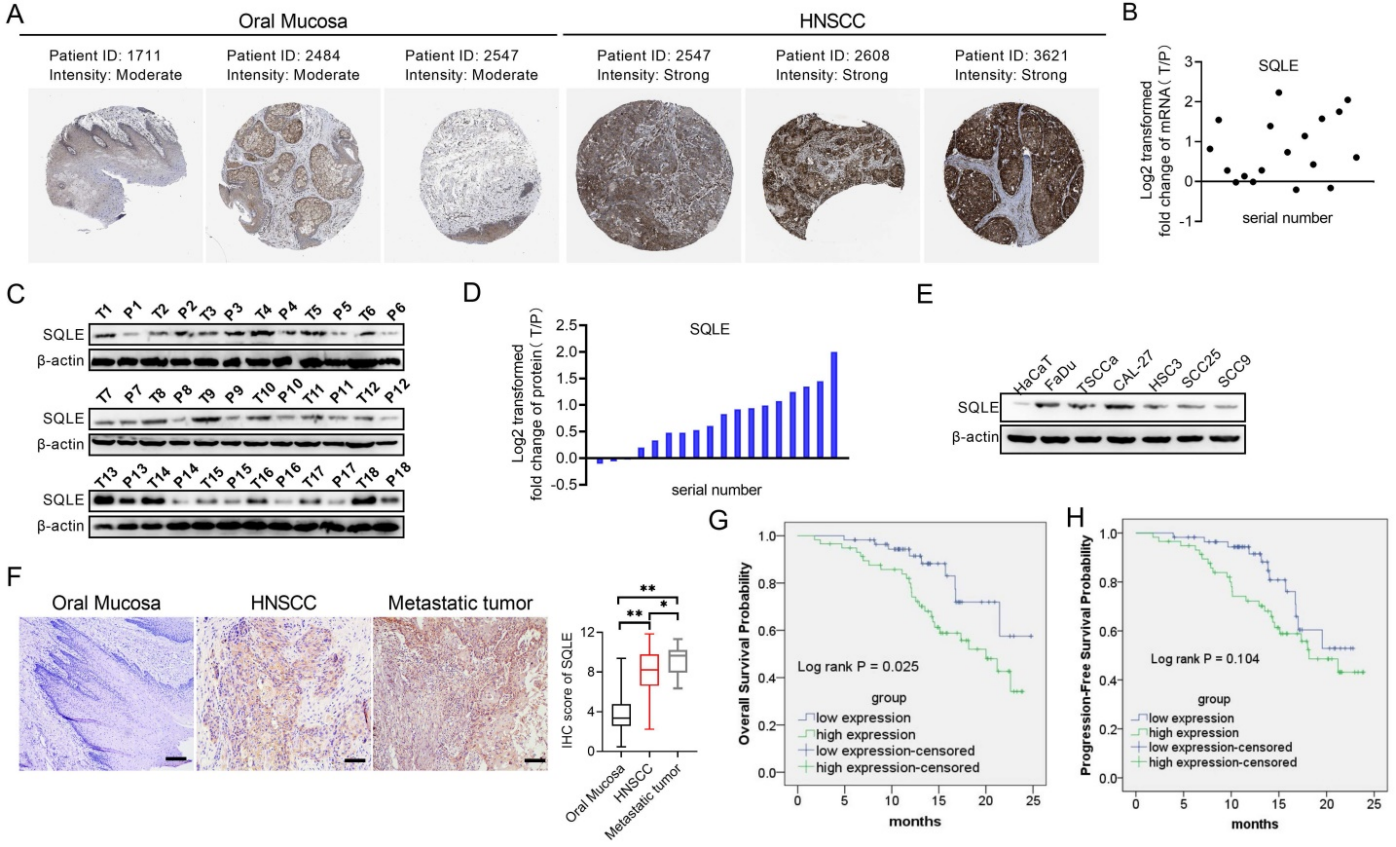
** SQLE expression is frequently overexpressed in HNSCC tissues based on experimental evidence.** (A) Analysis of SQLE protein level in oral mucosa (left panel) and HNSCC tissues (right panel) from HPA database. (B) PCR analysis of SQLE mRNA expression in HNSCC and peritumor tissues from 18 patients. (C-D) SQLE protein expression based on HNSCC and peritumor tissues from 18 patients. (E) Expression of SQLE in 6 HNSCC cell lines compared with HaCaT cell line. (F) IHC analysis of SQLE expression in primary HNSCC, metastatic HNSCC and peritumor tissues from our cohort. (G-H) Kaplan-Meier analyses for overall survival (OS) and progression-free survival (PFS) from our cohort. Abbreviation: HNSCC, head and neck squamous cell carcinoma.

To assess the role of SQLE in human HNSCC, we firstly explored SQLE expression in Human Protein Atlas (HPA) dataset and found SQLE expression was overexpressed in tumor tissues compared with peritumor tissues (Figure 2A). Next, we also performed qPCR and WB assays to determine the mRNA and protein expression levels of SQLE in our own cohort (n=18). Our results showed that SQLE was significantly upregulated in HNSCC tissues compared with peritumor tissues (Figures 2B-D). Also, compared with HaCaT cells, the expression of SQLE was also high in five HNSCC cells (Figure 2E). As shown in Figure 2F, IHC staining analysis in HNSCC from our cohort of 115 patients further confirmed the SQLE upregulation compared with peritumor samples. Also, we found SQLE expression was higher in 21 lymph nodal metastases than that in primary HNSCC lesions (Figure 2F). Finally, we explored the clinical relevance of SQLE protein expression. Kaplan-Meier survival analysis demonstrated that the overall survival (OS) and progression-free survival (PFS) of patients with high SQLE expression were notably shorter than that of patients with low SQLE expression (Figures 2G-H).

More importantly, high SQLE protein expression in HNSCC was closely associated with tumor size, distant metastasis, and advanced TNM stage, indicating that SQLE contribute to the progression of HNSCC (Table 2). Additionally, SQLE protein expression, distant metastasis, and TNM stage were assessed to be related with OS and PFS in univariate analysis (Table 3). Multivariate analysis depicted that SQLE expression could be a prognostic factor for OS and PFS of HNSCC participants after adjusting for distant metastasis and TNM stage, demonstrating that SQLE may be an independent prognostic factor for HNSCC (Table 4).

### SQLE upregulation is mediated by the downregulation of miR-584-5p in HNSCC

**Figure 3:**
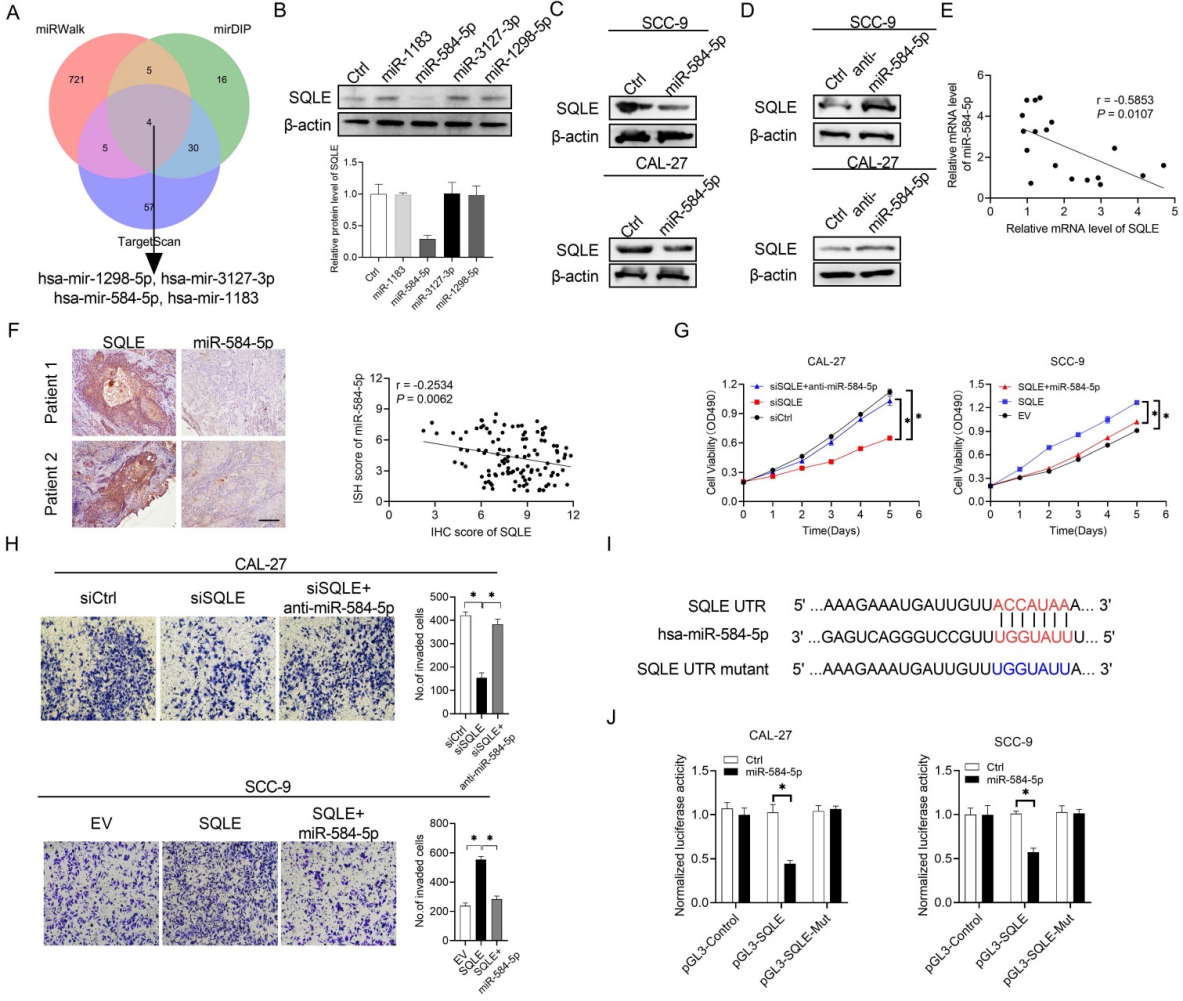
** SQLE upregulation is mediated by the downregulation of miR-584-5p in HNSCC.** (A) The intersection of three predicted target miRNA datasets. (B) Western blot analysis for SQLE expression in CAL-27 cells transfected with four predicted miRNAs targeting SQLE. (C and D) WB assay for determination of SQLE expression in HNSCC cells treated as indicated. (E) Relationship of miR-494-3p level with SQLE level in tumor tissues 18 paired HNSCC patients. (F) Expression analysis between SQLE protein level and miR-584-5p level in patients' samples. (G) MTS assay in CAL-27 and SCC-9 cells treated as specified. (H) Matrigel invasion assay in HNSCC cells treated as specified. (I-J) Dual luciferase reporter assay to determine regulating relationship of miR-584-5p on SQLE mRNA.

Substantial evidence has suggested that frequently dysregulated microRNA (miRNA) in cancers is important for regulating gene expression. In the study, three databases in predicting target programs were used to explore the latent miRNAs involved in the upregulation of SQLE in HNSCC. The four potential miRNAs were listed in Figure 3A. We explored miR-584-5p expression levels between paratumor and tumor groups via ISH technology, which the results showed miR-584-5p levels were higher in paratumor oral mucosa than them in HNSCC lesions, but the OS and PFS prognosis of HNSCC patients could not be well distinguished based on miR-584-5p expression level (Figure S1A-B). WB showed that miR-584-5p remarkably decreased SQLE expression in CAL-27A (Figures 3B-C) and SCC9 cells (Figures 3C), with transfection effects showing in Figure S1C. Conversely, miR-584-5p inhibition elevated the expression of SQLE (Figure 3D). Together, our results in HNSCC tissue detection found that mRNA level between SQLE and miR-584-5p existed significantly negative correlation (Figure 3E), which were furtherly supported by the negative correlation of SQLE protein level and miR-584-5p level (Figure 3F). Additionally, miR-584-5p mimics reduced the ability of SQLE to promote HNSCC cells proliferation, while miR-584-5p inhibitor treatment augmented the proliferation by SQLE overexpression in HNSCC cells (Figure 3G), with the corresponding transfection effects showing in Figure S1D-F. In addition, transwell invasion assay showed that miR-584-5p mimics attenuated the invasion of HNSCC cells mediated by SQLE, whereas miR-584-5p inhibition reversed this phenotype (Figure 3H). Altogether, these results suggest that miR-584-5p may lead to SQLE downregulation and abrogates the proliferation and invasion of HNSCC cells by inhibiting SQLE. To further determine the relationship between miR-584-5p and SQLE, we conducted dual luciferase reporter assay (Figure 3I). The results revealed that miR-584-5p significantly attenuated the luciferase activity of SQLE wild-type reporters compared with that of mutant reporters (Figure 3J). Altogether, these results suggest that downregulation of miR-584-5p may lead to SQLE upregulation and promote the proliferation and invasion of HNSCC cells.

### SQLE promotes HNSCC cell growth and metastasis *in vitro*

**Figure 4:**
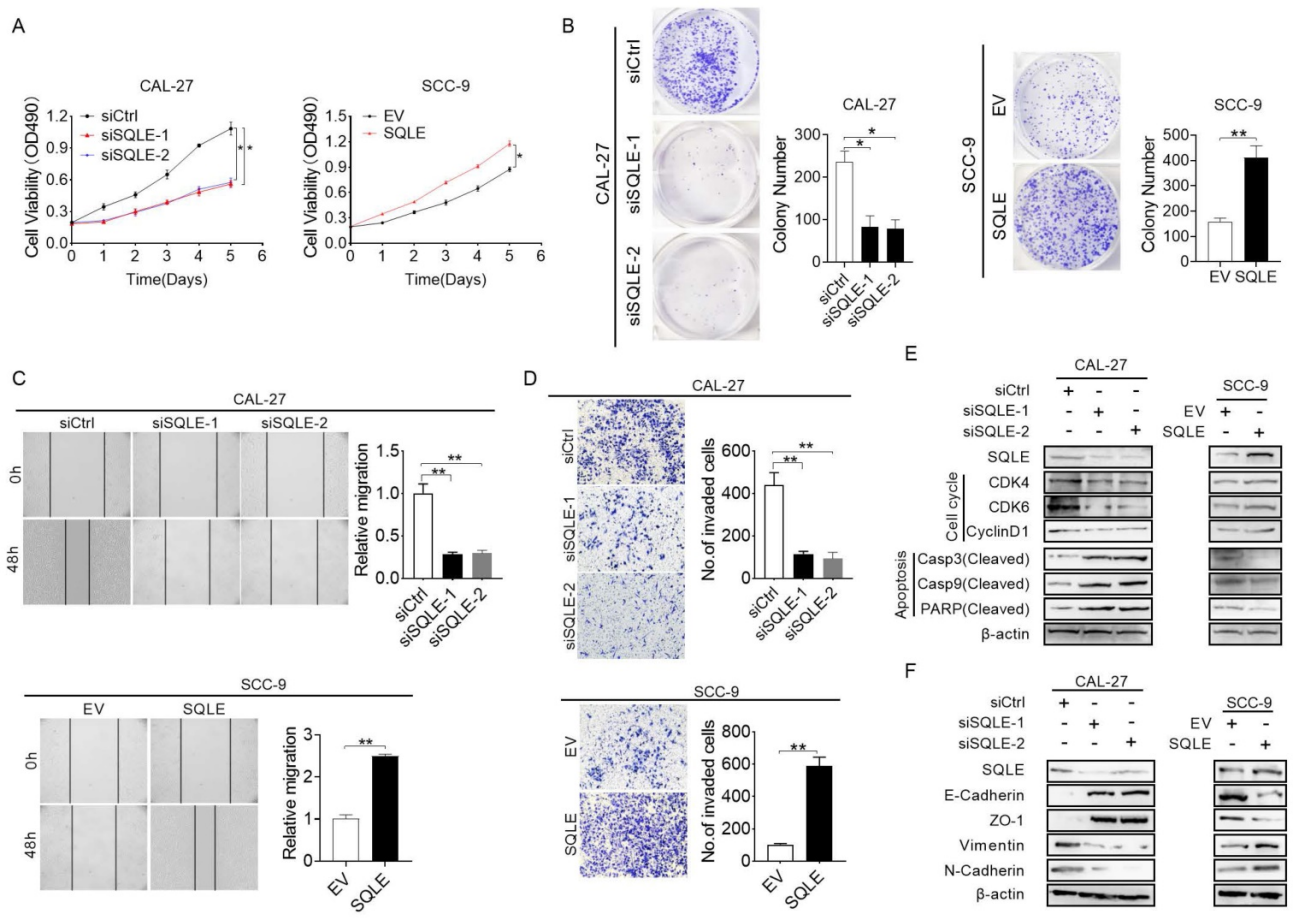
** SQLE upregulation promoted HNSCC cells proliferation and metastasis *in vitro*.** (A-B) MTS and colony formation assays for determining the cell growth ability of HNSCC cell treated as indicated. (C-D) Wound healing and transwell invasion assays for evaluating the cell migration ability of CAL-27 and SCC-9 cells treated as specified. (E) Expression of markers related to cell proliferation and apoptosis detected in HNSCC cells by WB assessment. (F) WB analysis of EMT-related protein expression in HNSCC cells treated as indicated.

To explore the function of SQLE in cell growth, we performed a series of *in vitro* experiments. The MTS assay revealed that SQLE knockdown in CAL-27 and FaDu cells significantly inhibited cell growth rate compared with the control group, while the effects of SQLE knockdown were markedly reversed by SQLE forced expression (Figure 4A and Figure S2A). Consistent with this, the number of colonies were significantly decreased in CAL-27 and FaDu cells after knockdown of SQLE, whereas forced expression of SQLE in SCC-9 and SCC-25 cells promoted cell growth (Figure 4B and Figure S2B). Furtherly, we conducted would closure and transwell assays to assess the function of SQLE in cell migration and invasion. The wound healing and transwell invasion assays revealed that SQLE knockdown attenuated the metastatic ability of CAL-27 and FaDu cells. Conversely, the metastatic ability of SCC-9 and SCC-25 cells with SQLE overexpression was greater than that of control group (Figures 4C-D and Figure S2C-D). In addition, to determine the mechanism of SQLE, we performed WB analysis to explore several markers related to cell growth and metastasis. As shown in Figure 4E and Figure S2E, the expression of major regulators of G1-S checkpoint (cyclin D1, cyclin-dependent kinases (CDK4 and CDK6)) was downregulated in CAL-27 and FaDu cells with SQLE knockdown, while SQLE upregulation in SCC-9 and SCC-25 cells had opposite effect, demonstrating function of SQLE in promoting cell cycle progression in HNSCC cells. Considering the pivotal role of epithelial-mesenchymal transition (EMT) in tumor metastasis, we then investigated the involvement of EMT in SQLE-mediated HNSCC metastasis. WB results showed SQLE silencing significantly inhibited epithelial-mesenchymal transition, which was evidenced by upregulation of epithelium regulators (E-cadherin and ZO-1) and downregulation of mesenchymal regulators (Vimentin and N-cadherin). And overexpression of SQLE remarkably reversed the effects of SQLE knockdown (Figure 4F and Figure S2F). Altogether, these above results indicate that SQLE boosts the proliferation and invasion of HNSCC cells.

### SQLE promotes HNSCC cell growth and metastasis *in vivo*

**Figure 5:**
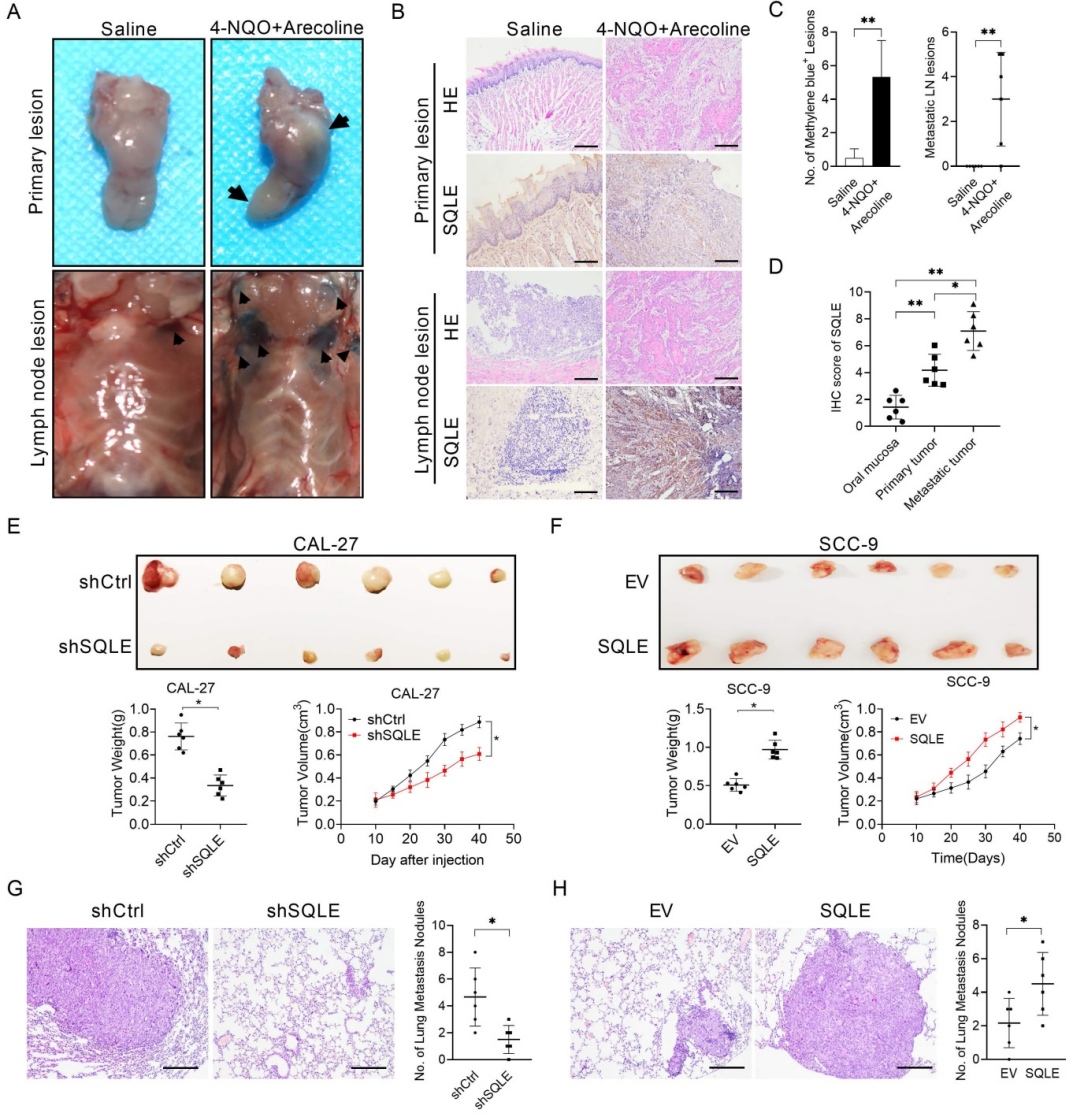
** SQLE upregulation promoted HNSCC cells proliferation and metastasis *in vivo*.** (A) Macroscopic examination of murine primary HNSCC and lymphoid metastatic lesions induced by 4-NQO and arecoline. (B) HE and IHC analyses of primary and metastatic HNSCC lesions treated as indicated. (C) No. of lymph node metastases treated as indicated. (D) IHC score of primary and metastatic HNSCC tissues compared with para-tumor tissues. (E) SQLE knockdown hindered growth of CAL-27 xenograft tumors, which were generated by injecting CAL-27-shSQLE cells or CAL-27-control cells. The growth of xenograft tumors was measured by volume, and the tumor weight was measured. (F) SQLE overexpression promoted growth of SCC-9 xenograft tumors, which were generated by injecting SCC-9 cells overexpressing SQLE or carrying an empty vector. The growth of xenograft tumors was measured by volume, and the tumor weight was measured. (G-H) SQLE knockdown hindered pulmonary metastases induced by tail vein injection of CAL-27 cells, while SQLE upregulation increased pulmonary metastases induced by tail vein injection of SCC-9 cells.

To explore the malignant function of SQLE *in vivo*, we performed a series of *in vivo* experiments. We conducted induced HNSCC tumorigenesis model using 4-nitroquino⁃line-l-oxide (4-NQO) (200 g/mL) and arecoline (500 g/mL) in drinking water for 16-20 weeks. To investigate lymph nodal metastasis, we used 0.1 mL methylene blue injection around tumor lesions under chloral hydrate-induced mice anesthesia state and together histological examination as conformations. Subsequent histological examination verified that SQLE was upregulated in tumor tissues compared with paired normal tissues, and SQLE was even higher in metastatic lymph node than that in primary tumors, which was similar with the results from human samples (Figure 5A-D). Also, we performed subcutaneous tumor growth assays using HNSCC cells with different SQLE expression levels. The results revealed that SQLE knockdown in CAL-27 cells significantly inhibited tumor volume and tumor weight compared with the control group, while the effects of SQLE knockdown were markedly reversed by SQLE forced expression (Figure 5E-F). Also, we investigated SQLE expression, Ki67 IHC staining score and TUNEL apoptotic cells proportion. The results showed knockdown of SQLE decreased Ki67^+^-cells rate and increased apoptotic cell proportion, while SQLE overexpression reversed the phenotypes of SQLE knockdown (Figure S3). Consistent with this, the number of pulmonary metastases caused by tail vein injection of tumor cells were significantly decreased in CAL-27 cells after knockdown of SQLE, whereas forced expression of SQLE in SCC-9 cells promoted tumor metastasis (Figure 5G-H). Altogether, these above results indicate that SQLE promotes the tumor growth and metastasis of HNSCC cells *in vivo*.

### Correlation and enrichment analyses

**Figure 6:**
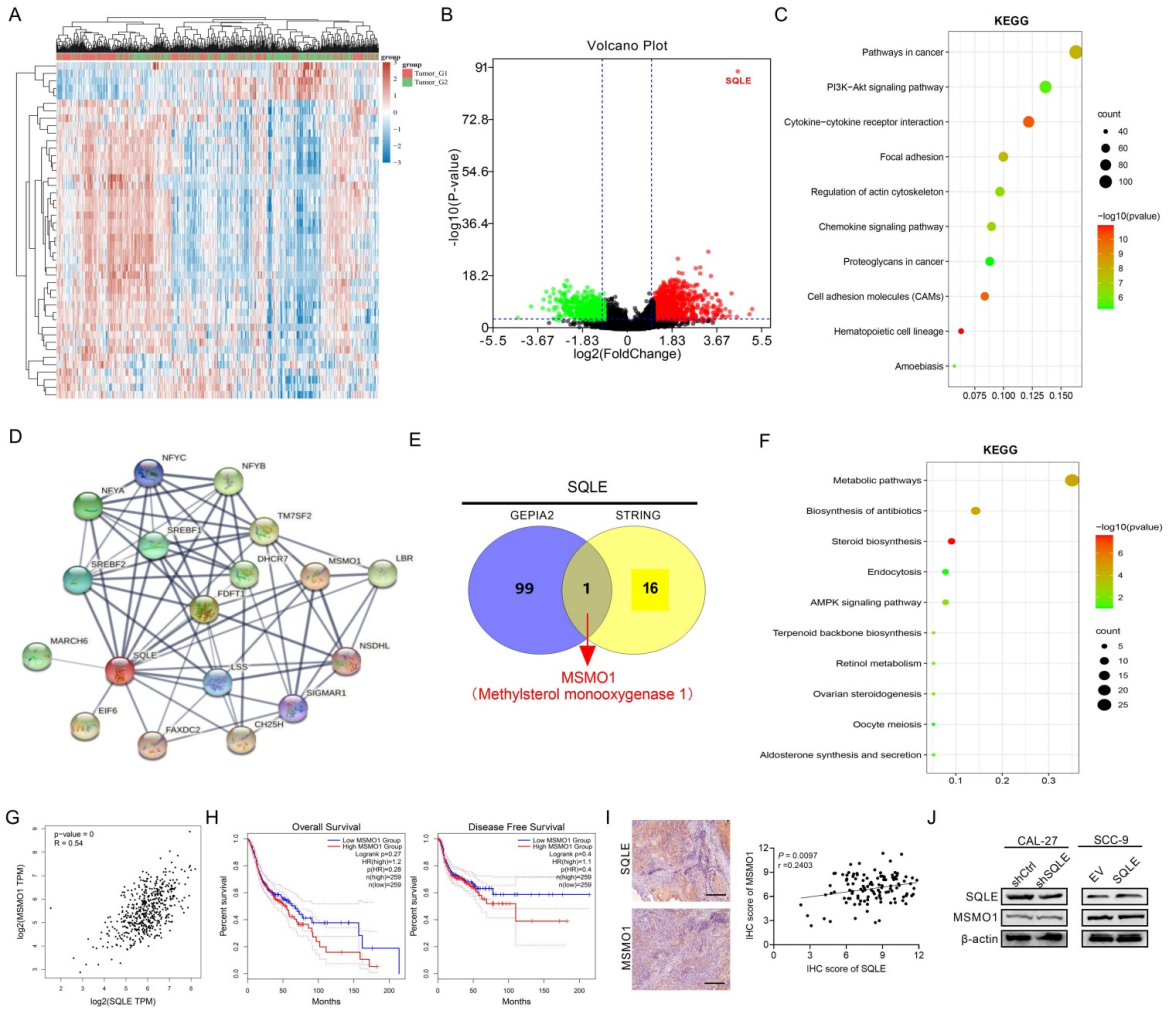
** SQLE was associated with cholesterol biosynthesis and PI3K/Akt signaling activation.** (A-B) Heat map and volcano map showing SQLE-related different genes in TCGA-HNSCC samples. (C) Bubble chart to analyze the enrichment pathways related to SQLE expression from TCGA dataset. (D) STRING analysis showing the most related protein with SQLE. (E) The intersection between STRING and top 100 SQLE-related genes. (F) KEGG enrichment analysis from the union set of STRING and top related genes from GEPIA2. (G) The Spearman's rank correlation coefficient between SQLE and MSMO1 based on TCGA-HNSCC dataset. (H) Kaplan-Meier assessment for OS and PFS from GEPIA2. (I) The Spearman's rank correlation coefficient between SQLE and MSMO1 according to our cohort. (J) WB analyses of MSMO1 expression levels treated by shSQLE or SQLE in CAL-27 or SCC-9 cells. Abbreviation: KEGG, Kyoto Encyclopedia of Genes and Genomes.

To predict the function of SQLE, including associated pathways, we performed a correlation analysis between SQLE and other genes in HNSCC using TCGA data (Figures 6A-B). We explored the potential functional pathways according to the cut-off points of *p*-value<0.01 and |logFC|>2 using DAVID database (https://david.ncifcrf.gov/summary.jsp). Kyoto Encyclopedia of Genes and Genomes (KEGG) pathway analysis revealed that SQLE was primarily associated with PI3K/Akt pathway terms (Figures 6C). Moreover, we further explored the potential functional pathways based on the STRING database (Figure 6D). And methylsterol monooxygenase 1 (MSMO1) was screened via taking the intersection between STRING and top 100 related genes of GEPIA2 (http://gepia2.cancer-pku.cn/#general)^22^ (Figure 6E). Also, KEGG pathway analysis showed that SQLE was primarily related to metabolic and cholesterol biosynthesis pathway terms according to the union set of STRING and top 100 related genes of GEPIA2 (Figures 6F). Besides, we preliminarily explored MSMO1 functions in HNSCC. Although the mRNA levels between SQLE and MSMO1 are very positively correlative, MSMO1 expression did not seem to predict the OS and DFS prognosis of patients and further the protein levels between the two showed weakly negative correlation (Figure 6G-I). We also examined MSMO1 expression after knockdown or overexpression of SQLE, and the results showed MSMO1 expression did not show significant differences (Figure 6J). These results suggest that SQLE is associated with various cancer-related pathways in HNSCC, especially cholesterol metabolic and PI3K/Akt pathways.

### PI3K/Akt pathway is involved in SQLE-mediated HNSCC cells malignant phenotypes

Based on enrichment analysis that cholesterol biosynthesis signaling and activation of PI3K/Akt pathway were involved in tumor growth and metastasis, we hypothesized the phenotypes induced by SQLE upregulation may be relevant with cholesterol content and PI3K/Akt pathway activation.

**Figure 7:**
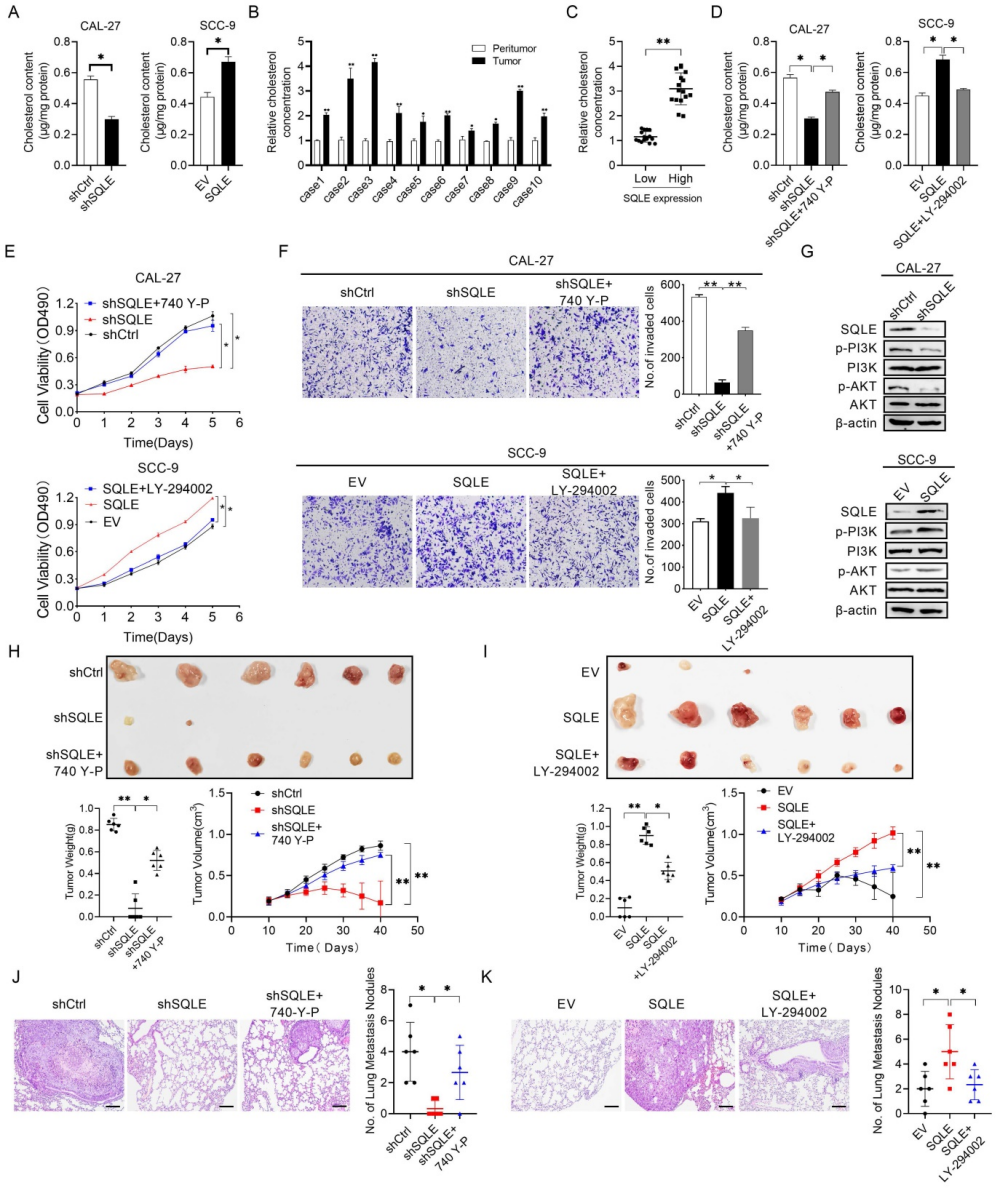
** SQLE elevated intracellular cholesterol and activated PI3K/Akt signaling.** (A) Cholesterol content assessment in CAL-27 and SCC-9 cells treated as indicated. (B) Cholesterol content in 10 paired HNSCC and peritumor samples. (C) Correlation between SQLE expression and cholesterol concentration in HNSCC tissues. (D) Cholesterol content assay in CAL-27 and SCC-9 cells treated by 740 Y-P and LY-294002. (E-F) MTS and transwell invasion experiments for determining the cell proliferation and invasion ability of HNSCC cells treated as indicated. (G) WB analysis for assessing PI3K/Akt signaling activation in HNSCC cells treated as specified. (H-I) The growth analyses of xenograft tumors treated as indicated. (J-K) Analyses of pulmonary metastatic lesions treated as indicated.

To verify this possibility, we first measured cholesterol content in stable SQLE knockdown CAL-27 cells and SQLE overexpression SCC-9 cells. Indeed, SQLE knockdown decreased the levels of intracellular cholesterol compared with the control cells and SQLE overexpression increased cholesterol level compared with empty vector group (Figure 7A). Moreover, the concentration of cholesterol in HNSCC tumor tissues was higher than that in the paired peritumor tissues (Figure 7B). Together, SQLE ^high^ HNSCC tumors had high levels of cholesterol in comparison with SQLE ^low^ tumors (Figure 7C). These results suggest that SQLE mediated cholesterol increase plays vital role in HNSCC malignancy. Next, a strong relationship between cholesterol and PI3K/Akt pathway activation was demonstrated by a series of studies 5, 23, 24, we treated CAL-27 cells or SCC-9 cells with PI3K/Akt signaling agonist 740 Y-P or PI3K/Akt signaling antagonist LY294002. As shown in Figure 7D, 740 Y-P treatment significantly increased cholesterol concentration of CAL-27 cells induced by SQLE knockdown, whereas LY294002 treatment significantly decreased cholesterol level of SCC-9 cells enhanced by SQLE overexpression.

Also, the results of MTS cell viability assay and transwell matrigel invasion assay also showed that 740 Y-P increased the growth and invasion of CAL-27 cells induced by SQLE knockdown, whereas LY294002 significantly abrogated the growth and invasion of SCC-9 cells promoted by SQLE overexpression, suggesting PI3K/Akt signaling is involved in the SQLE upregulation-mediated HNSCC tumor growth (Figures 7E-F). As expected, western blot analysis showed that SQLE knockdown in CAL-27 cells decreased the protein expression of p-PI3K, p-Akt, while overexpression of SQLE in SCC-9 cells had the opposite effect (Figure 7G). Importantly, considering robust modulating role of PI3K/Akt signaling in cells, we tested whether PI3K/Akt signaling could affect SQLE expression. And the results showed 740 Y-P treatment increased SQLE expressions, whereas LY294002 treatment decreased them (Figure S4A-B). Together, the result suggests SQLE and PI3K/Akt formed positive feedback and SQLE-mediated cholesterol increase is dependent on PI3K/Akt signaling activation in HNSCC cells. Last but not the least, the results of in vivo tumor growth and metastasis also showed that 740 Y-P increased the tumor growth and pulmonary metastases of CAL-27 cells induced by SQLE knockdown, whereas LY294002 significantly abrogated the tumor growth and pulmonary metastases of SCC-9 cells promoted by SQLE overexpression, demonstrating that SQLE promotes HNSCC tumor growth and metastasis via activating PI3K/Akt signaling (Figure 7H-K and Figure S4C-H). These results confirm the role of SQLE in promoting malignant behaviors in HNSCC cells by activating the PI3K/Akt pathway.

### Terbinafine could inhibit HNSCC cell growth and metastasis *in vitro*

**Figure 8:**
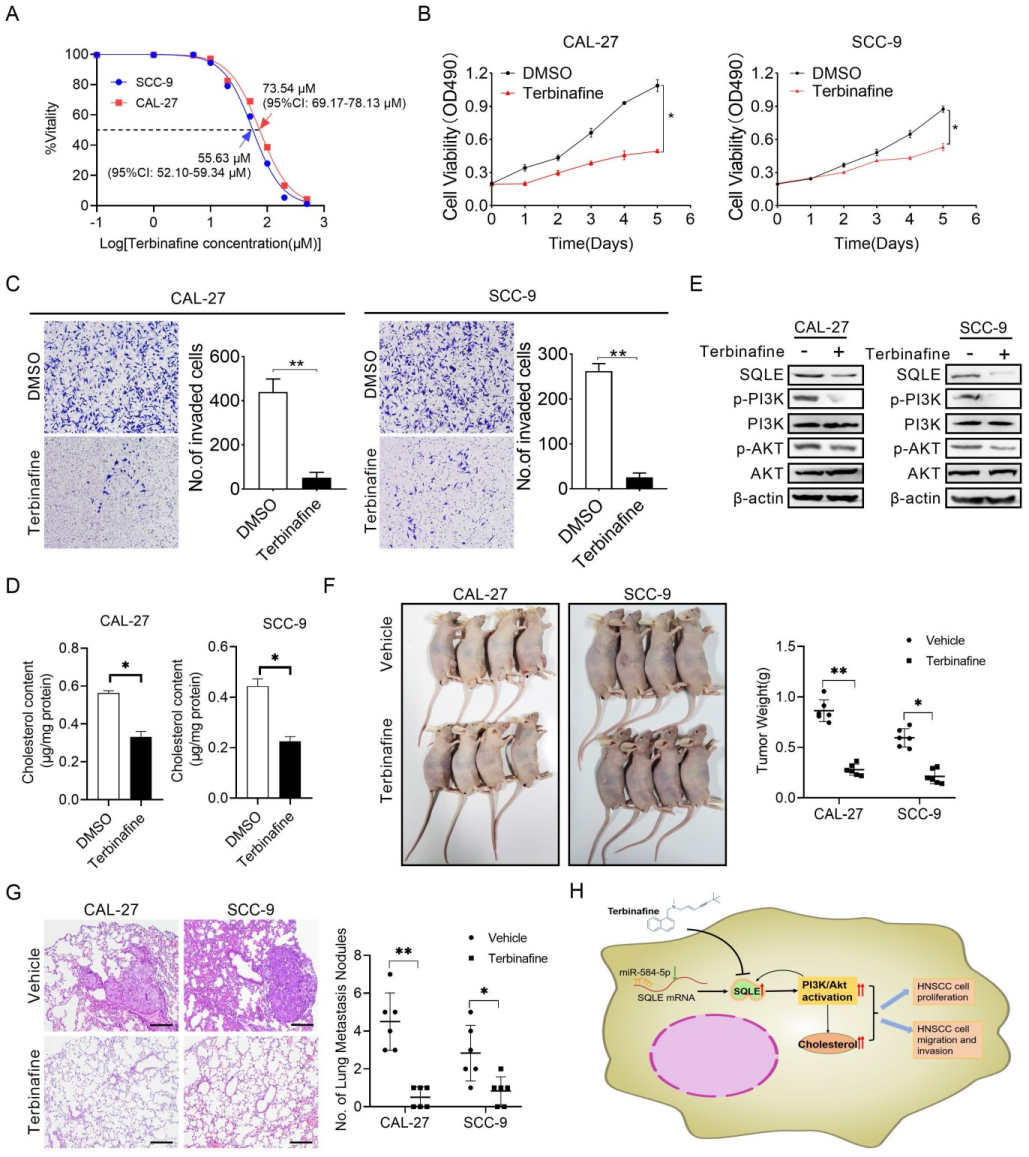
** Terbinafine exhibited a therapeutic effect on HNSCC *in vitro*.** (A) IC50 values of CAL-27 and SCC-9 cells treated by terbinafine. (B-C) MTS, as well as matrigel invasion assessments were performed in HNSCC cells exposed to terbinafine. (D) Intracellular cholesterol evaluation in CAL-27 and SCC-9 cells treated with terbinafine. (E) WB analysis for assessing PI3K/Akt pathway interference in CAL-27 and SCC-9 cells exposed to terbinafine. (F) The growth analyses of xenograft tumors treated as indicated. (G) Analyses of pulmonary metastatic lesions treated as indicated. (H) Graphic abstract of this study.

Terbinafine is a selectively antagonist of SQLE, which has been used in the treatment of fungal infections for more than 20 years 25. To study the potential therapeutic effect of terbinafine in HNSCC, we firstly assessed the effect of terbinafine on the ability of SQLE to promote cell growth and metastasis. As shown in Figure 8A, the IC50 of terbinafine treatment on CAL-27 and SCC-9 cells were 73.54 μM (95%CI: 69.17-78.13 μM) and 55.63 μM (95%CI: 52.10-59.34 μM) respectively, with significant difference between the two IC50 values. Furtherly, terbinafine treatment markedly decreased HNSCC cell growth rate in CAL-27A and SCC-9 cells (Figures 8B). Treatment with terbinafine remarkably decreased invaded cell numbers in CAL-27A and SCC-9 cells (Figures 8C). Also, cholesterol content in CAL-27 and SCC-9 cells was reduced by terbinafine treatment (Figures 8D). Notably, terbinafine treatment remarkably inhibited SQLE expression and PI3K/Akt signaling (Figures 8E). Furtherly, terbinafine treatment (80 mg/kg, oral daily) significantly decreased tumor growth and pulmonary metastases of HNSCC cells (Figure 8F-G). Also, we investigated SQLE expression, Ki67 IHC staining score and TUNEL apoptotic cells proportion. The results showed terbinafine treatment decreased Ki67^+^-cells rate and increased apoptotic cell proportion (Figure S5). These data imply that reduced expression of SQLE by terbinafine may be a prospective novel therapeutic approach for HNSCC.

## Discussion

Recently, SQLE upregulation was reported and identified as a prognostic marker in a variety of disease, such as non-alcoholic steatohepatitis 26, hepatocellular carcinoma 27 and breast cancer 28. In the present study, as shown in Figure 8H, we investigated the function of SQLE in HNSCC and found that SQLE was upregulated in tumor tissues compared with paracancerous tissues. Moreover, SQLE overexpression was associated with poor prognosis of HNSCC patients. Further analysis indicated that high SQLE expression level in HNSCC was significantly associated with distant metastasis and TNM stage, indicating that SQLE may be involved in HNSCC progression. Mechanistically, overexpression of SQLE in HNSCC was induced by the downregulation of miR-584-5p. Functional study revealed that SQLE promoted tumor growth and invasion *in vitro*. Moreover, terbinafine, a selective inhibitor of SQLE, abrogated the growth and invasion of HNSCC cells, providing a candidate drug for HNSCC treatment.

It has been reported that SQLE participated in the regulation of tumor development and progression. Liu et al. preliminarily reported SQLE upregulation in HNSCC cells might predict poor prognosis 7. To our knowledge, it is the first study to illustrate the functional mechanisms of SQLE in HNSCC. To determine the biological role of SQLE in HNSCC, we conducted bioinformatic analysis of gene function in HNSCC tissues, and the results revealed that SQLE promoted cholesterol metabolism and PI3K/Akt signal. Next, *in vitro* and *in vivo* experiments confirmed that SQLE significantly aggregated the proliferation and invasion of HNSCC cells via increasing intracellular cholesterol and activating PI3K/Akt signaling. However, the study is still relatively preliminary, and more* SQLE* gene knockout assays should be investigated to verify the function and molecular mechanism of SQLE promoting HNSCC progression.

SQLE is one of the rate-limiting enzymes in cholesterol biosynthesis. Cholesterol metabolism is essential for tumor growth. Many cancer cell lines depend on exogenous cholesterol for their growth. For example, when lacking lipoprotein in serum, histiocytic lymphoma cells and clear cell renal cell carcinoma (ccRCC) cells die unless supplemented with exogenous cholesterol 29, 30. In addition, lower levels of LDLR but higher levels of SQLE are expressed in advanced-stage prostate cancer, revealing a greater dependence on endogenous cholesterol synthesis than uptake 31. However, in colorectal cancer (CRC), excess cholesterol which might be induced by up-regulation of LDLR on CRC cells could degrade SQLE protein and low expression of SQLE could promote CRC malignancy 32. Together, we evaluated the mRNA expressions of key regulators of cholesterol metabolic flux in TCGA-HNSCC dataset and found uptake regulators (LDLR, LOX1, LRP1, SCARB1), biosynthesis regulators (SQLE, DHCR7) and efflux regulators (ABCA1, ABCG1) were upregulated and bioconversion regulators (CYP11A1, CYP27A1) were deregulated in HNSCC tumor tissues compared with paracancerous tissues (data not shown). And thus, it indicates intracellular cholesterol in cancer cells elevate but sustain homeostasis status. Besides, though MSMO1 expression showed significantly positive correlation with SQLE, its protein level exhibited weakly positive correlation. The paradox might be attributed to complicated post-transcriptional and post-translational modulating process which is needed to be evaluated in the future. Not coincidentally, a recent report has indicated that MSMO1 might contribute to stemness maintenance of nasopharyngeal carcinoma cells 33. And thus, it might be valuable to investigate the role of MSMO1 in HNSCC and even varieties of cancers in the future. As above, many tumor cells rely on cholesterol to promote tumor growth. Moreover, cholesterol has been demonstrated to regulate several oncogenous signals, such as PI3K/Akt 23 and Wnt/β-catenin 34. Our study was only based on bioinformatic analysis and verified the hypothesis of the involvement of PI3K/Akt pathway in HNSCC progression. Though bias existed in the study, our results demonstrated PI3K/Akt is involved in SQLE-mediated cholesterol synthesis and tumor progression, which was consistent with the conclusions of other studies. Also, Zhang et al. reported terbinafine significantly inhibited HCC cells proliferation by suppressing mTORC1 signaling 27. In our results based on KEGG pathway analysis, AMPK signaling was enriched, suggesting complicated relationship between AMPK and PI3K/Akt/mTOR signaling involve in SQLE-mediated HNSCC progression. Moreover, KEGG analysis showed SQLE was closely related with cell-cell interaction. Also, Liu et al. illustrated SQLE expression was negatively associated with the infiltration of CD8+ T cells, follicular helper T cells, and regulatory T cell infiltration and mast cell activation in HNSCC, indicating SQLE function important role in regulating tumor microenvironment (TME) and more experimental evidence might be given 7. Therefore, SQLE may promote the progression of HNSCC through various mechanisms, which needs to be furtherly determined in future work.

MicroRNAs regulate target mRNAs at the post-transcriptional level by resulting in degradation of target mRNAs 35. MiR-584-5p is a frequently downregulated factor and is involved in the growth, metastasis, and chemoresistance in a variety of cancers. For example, Abdelfattah et al. demonstrated that miR-584-5p inhibited medulloblastoma growth in pre-clinical tumor models and potentiates medulloblastoma to radiation and vincristine 36. Li et al. reported that downregulation of miR-584-5p aggregated tumor progression of gastric cancer via targeting WW domain-containing E3 ubiquitin protein ligase 1^37^. In our study, miR-584-5p is also downregulated in HNSCC and negatively correlated with SQLE expression, which inhibits HNSCC progression. Moreover, we found that miR-584-5p inhibited HNSCC cell growth and invasion by antagonizing SQLE. Thus, miR-584-5p/SQLE axis might be a prospective strategy for HNSCC treatment. What's more, we still cannot rule out the possibility that other factors may also contribute to the upregulation of SQLE in HNSCC cells, such as genetic and epigenetic aberrations. Therefore, the possibility of involvement of other factors in SQLE overexpression in HNSCC cells still needs further confirmation.

Terbinafine is a selectively antagonist of SQLE, which has been widely utilized in treating fungal infections. A previous study has indicated that terbinafine could inhibit tumor progression of breast cancer 28. Also, Zhang et al. reported terbinafine intensely alleviated the growth of HCC cells by suppressing mTORC1 signaling 27. However, the potential application of SQLE inhibitors to treat HNSCC has not been explored until now. Our results clearly showed terbinafine treatment significantly suppressed the proliferation and invasion of HNSCC *in vitro* and* in vivo*, suggesting that inhibition of SQLE by terbinafine may be a promising therapeutic approach to the treatment of HNSCC. Particularly, we should point out that the results are far from comprehensive to apply to preclinical and clinical practice.

In summary, this study shows that up-regulated SQLE promotes both the growth and metastasis of HNSCC cells via increasing intracellular cholesterol biosynthesis and activating PI3K/Akt signaling. Moreover, terbinafine treatment significantly suppresses HNSCC cells growth and invasion. These findings suggest that SQLE plays a key oncogenous function in HNSCC and may be a potential therapeutic target for HNSCC treatment.

## Supplementary Material

Supplementary figures.

## Figures and Tables

**Table 1:** Details of GEO series included in this analysis

GEO series	Contributor(s), Year	Tumor	Non-tumor	Platform
GSE33232	Stansfield JC, 2016	25	50	[HuEx-1_0-st] Affymetrix Human Exon 1.0 ST Array [transcript (gene) version]
GSE59102	Bueno RB, 2014	132	29	Agilent-014850 Whole Human Genome Microarray 4x44K G4112F (Probe Name version)
GSE107591	Blandino G, 2017	22	22	[HuGene-1_0-st] Affymetrix Human Gene 1.0 ST Array [transcript (gene) version]
GEO series	Contributor(s), Year	Primary	Metastatic	Platform
GSE136037	De Cecco L, 2020	47	25	Illumina HumanHT-12 WG-DASL V4.0 R2 expression beadchip

**Table 2:** Correlation between tumor SQLE expression and clinicopathologic features.

Variables	No. of cases (%)	SQLE expression		*P* value
		Low	High	
All	115(100%)	62	53	
Age				0.154
<60	59	28	31	
>=60	56	34	22	
Gender				0.435
Female	37	18	19	
Male	78	44	34	
Tobacco				0.873
No	53	29	24	
Yes	62	33	29	
Alcohol				0.110
No	58	27	31	
Yes	57	35	22	
HPV				0.165
Negative	96	49	47	
Positive	19	13	6	
Tumor size				**0.004**
<4cm	49	34	15	
>=4cm	66	28	38	
Differentiation grade				0.207
Well	71	35	36	
Poor	44	27	17	
Lymph node metastasis				0.187
Absent	51	31	20	
Present	64	31	33	
Distant metastasis				**0.024**
Absent	72	33	39	
Present	43	29	14	
TNM stage				**0.009**
I+ II	92	44	48	
III+ IV	23	18	5	

**Table 3:** Association of SQLE and clinical factors with overall survival

	Unadjusted HR * (95% CI)	P	Adjusted HR† (95% CI)	P
SQLE expression	1.382(1.119-2.421)	**0.000**	1.304(1.014-2.546)	**0.000**
Alcohol	0.994(0.849-9.471)	0.712	-	-
Age	1.027(0.088-10.241)	0.981	-	-
HPV	1.079(0.723-6.774)	0.712	-	-
Lymph node metastasis	0.911(0.433-10.531)	0.931	-	-
Distant metastasis	1.627(1.283-9.924)	**0.002**	2.3277(1.283-9.924)	**0.000**
Tumor size	1.507(0.497-15.315)	0.357	-	-
TNM stage	43.259(4.291-453.103)	**0.001**	88.045(11.323-734.436)	**0.000**

^*^Hazard ratios in univariate models ^†^Hazard ratios in multivariable models Abbreviations: HR, hazard ratio; 95% CI, 95% confidence interval.

**Table 4:** Association of SQLE and clinical factors with progression-free survival

	Unadjusted HR * (95% CI)	P	Adjusted HR† (95% CI)	P
SQLE expression	1.508(1.011-4.223)	**0.000**	0.006(0.002-0.022)	**0.000**
Alcohol	1.002(0.751-10.399)	0.769	-	-
Age	0.843(0.207-9.146)	0.715	-	-
HPV	1.220(0.462-12.613)	0.800	-	-
Lymph node metastasis	0.780(0.206-8.331)	0.407	-	-
Distant metastasis	2.106(1.455-13.174)	**0.000**	11.269(4.865-26.102)	**0.000**
Tumor size	1.186(0.843-55.913)	0.241	-	-
TNM stage	39.946(3.521-415.390)	**0.000**	0.006(0.002-0.022)	**0.000**

^*^Hazard ratios in univariate models ^†^Hazard ratios in multivariable models Abbreviations: HR, hazard ratio; 95% CI, 95% confidence interval.

## Abbreviations

SQLE: squalene epoxidase
HNSCC: head and neck squamous cell carcinoma
IHC: immunohistochemistry
WB: western blot
MTS: 3-(4,5-dimethylthiazol-2-yl)-5-(3-carboxymethoxyphenyl)-2-(4-sulfophenyl)- 2H-tetrazolium
OS: overall survival
PFS: progression-free survival
ISH: in situ hybridization
HR: hazard ratio
EMT: epithelial-mesenchymal transition
EV: empty vector
DMSO: dimethyl sulfoxide

## References

[1] Sun S, Wu Y, Guo W, Yu F, Kong L, Ren Y (2018). STAT3/HOTAIR Signaling Axis Regulates HNSCC Growth in an EZH2-dependent Manner. Clinical cancer research: an official journal of the American Association for Cancer Research.

[2] Liu C, Yu Z, Huang S, Zhao Q, Sun Z, Fletcher C (2019). Combined identification of three miRNAs in serum as effective diagnostic biomarkers for HNSCC. EBioMedicine.

[3] Takeda I, Maruya S, Shirasaki T, Mizukami H, Takahata T, Myers JN (2007). Simvastatin inactivates beta1-integrin and extracellular signal-related kinase signaling and inhibits cell proliferation in head and neck squamous cell carcinoma cells. Cancer science.

[4] Yang F, Kou J, Liu Z, Li W, Du W (2021). MYC Enhances Cholesterol Biosynthesis and Supports Cell Proliferation Through SQLE. Frontiers in cell and developmental biology.

[5] Li L, Zhang Q, Wang X, Li Y, Xie H, Chen X (2020). Squalene epoxidase-induced cholesteryl ester accumulation promotes nasopharyngeal carcinoma development by activating PI3K/AKT signaling. Cancer science.

[6] Qin Y, Hou Y, Liu S, Zhu P, Wan X, Zhao M (2021). A Novel Long Non-Coding RNA lnc030 Maintains Breast Cancer Stem Cell Stemness by Stabilizing SQLE mRNA and Increasing Cholesterol Synthesis. Advanced science (Weinheim, Baden-Wurttemberg, Germany).

[7] Liu Y, Fang L, Liu W (2021). High SQLE Expression and Gene Amplification Correlates with Poor Prognosis in Head and Neck Squamous Cell Carcinoma. Cancer management and research.

[8] Stansfield JC, Rusay M, Shan R, Kelton C, Gaykalova DA, Fertig EJ (2016). Toward Signaling-Driven Biomarkers Immune to Normal Tissue Contamination. Cancer informatics.

[9] Verduci L, Ferraiuolo M, Sacconi A, Ganci F, Vitale J, Colombo T (2017). The oncogenic role of circPVT1 in head and neck squamous cell carcinoma is mediated through the mutant p53/YAP/TEAD transcription-competent complex. Genome biology.

[10] Alfieri S, Carenzo A, Platini F, Serafini MS, Perrone F, Galbiati D (2020). Tumor Biomarkers for the Prediction of Distant Metastasis in Head and Neck Squamous Cell Carcinoma. Cancers.

[11] Rhodes DR, Yu J, Shanker K, Deshpande N, Varambally R, Ghosh D (2004). ONCOMINE: a cancer microarray database and integrated data-mining platform. Neoplasia (New York, NY).

[12] Gautier L, Cope L, Bolstad BM, Irizarry RA (2004). affy-analysis of Affymetrix GeneChip data at the probe level. Bioinformatics (Oxford, England).

[13] Ritchie ME, Phipson B, Wu D, Hu Y, Law CW, Shi W (2015). limma powers differential expression analyses for RNA-sequencing and microarray studies. Nucleic acids research.

[14] Chandrashekar DS, Bashel B, Balasubramanya SAH, Creighton CJ, Ponce-Rodriguez I, Chakravarthi B (2017). UALCAN: A Portal for Facilitating Tumor Subgroup Gene Expression and Survival Analyses. Neoplasia (New York, NY).

[15] Gao J, Aksoy BA, Dogrusoz U, Dresdner G, Gross B, Sumer SO (2013). Integrative analysis of complex cancer genomics and clinical profiles using the cBioPortal. Science signaling.

[16] Sticht C, De La Torre C, Parveen A, Gretz N (2018). miRWalk: An online resource for prediction of microRNA binding sites. PloS one.

[17] Tokar T, Pastrello C, Rossos AEM, Abovsky M, Hauschild AC, Tsay M (2018). mirDIP 4.1-integrative database of human microRNA target predictions. Nucleic acids research.

[18] Agarwal V, Bell GW, Nam JW, Bartel DP (2015). Predicting effective microRNA target sites in mammalian mRNAs. eLife.

[19] Huang da W, Sherman BT, Lempicki RA (2009). Systematic and integrative analysis of large gene lists using DAVID bioinformatics resources. Nature protocols.

[20] Szklarczyk D, Morris JH, Cook H, Kuhn M, Wyder S, Simonovic M (2017). The STRING database in 2017: quality-controlled protein-protein association networks, made broadly accessible. Nucleic acids research.

[21] Qiu Z, Liang N, Huang Q, Sun T, Xue H, Xie T (2020). Downregulation of DUSP9 Promotes Tumor Progression and Contributes to Poor Prognosis in Human Colorectal Cancer. Frontiers in oncology.

[22] Tang Z, Kang B, Li C, Chen T, Zhang Z (2019). GEPIA2: an enhanced web server for large-scale expression profiling and interactive analysis. Nucleic acids research.

[23] Mathews ES, Appel B (2016). Cholesterol Biosynthesis Supports Myelin Gene Expression and Axon Ensheathment through Modulation of P13K/Akt/mTor Signaling. The Journal of neuroscience: the official journal of the Society for Neuroscience.

[24] Yue S, Li J, Lee SY, Lee HJ, Shao T, Song B (2014). Cholesteryl ester accumulation induced by PTEN loss and PI3K/AKT activation underlies human prostate cancer aggressiveness. Cell metabolism.

[25] Liang QF, Jin XY, Wang XL, Sun XG (2009). Effect of topical application of terbinafine on fungal keratitis. Chinese medical journal.

[26] Liu D, Wong CC, Zhou Y, Li C, Chen H, Ji F (2021). Squalene Epoxidase Induces Nonalcoholic Steatohepatitis Via Binding to Carbonic Anhydrase III and is a Therapeutic Target. Gastroenterology.

[27] Zhang EB, Zhang X, Wang K, Zhang F, Chen TW, Ma N (2021). Antifungal agent Terbinafine restrains tumor growth in preclinical models of hepatocellular carcinoma via AMPK-mTOR axis. Oncogene.

[28] Brown DN, Caffa I, Cirmena G, Piras D, Garuti A, Gallo M (2016). Squalene epoxidase is a bona fide oncogene by amplification with clinical relevance in breast cancer. Scientific reports.

[29] Garcia-Bermudez J, Baudrier L, Bayraktar EC, Shen Y, La K, Guarecuco R (2019). Squalene accumulation in cholesterol auxotrophic lymphomas prevents oxidative cell death. Nature.

[30] Riscal R, Bull CJ, Mesaros C, Finan JM, Carens M, Ho ES (2021). Cholesterol auxotrophy as a targetable vulnerability in clear cell renal cell carcinoma. Cancer discovery.

[31] Stopsack KH, Gerke TA, Sinnott JA, Penney KL, Tyekucheva S, Sesso HD (2016). Cholesterol Metabolism and Prostate Cancer Lethality. Cancer research.

[32] Jun SY, Brown AJ, Chua NK, Yoon JY, Lee JJ, Yang JO (2021). Reduction of Squalene Epoxidase by Cholesterol Accumulation Accelerates Colorectal Cancer Progression and Metastasis. Gastroenterology.

[33] Zhang P, He Q, Wang Y, Zhou G, Chen Y, Tang L (2022). Protein C receptor maintains cancer stem cell properties via activating lipid synthesis in nasopharyngeal carcinoma. Signal transduction and targeted therapy.

[34] Sheng R, Kim H, Lee H, Xin Y, Chen Y, Tian W (2014). Cholesterol selectively activates canonical Wnt signalling over non-canonical Wnt signalling. Nature communications.

[35] Lee YS, Dutta A (2009). MicroRNAs in cancer. Annual review of pathology.

[36] Abdelfattah N, Rajamanickam S, Panneerdoss S, Timilsina S, Yadav P, Onyeagucha BC (2018). MiR-584-5p potentiates vincristine and radiation response by inducing spindle defects and DNA damage in medulloblastoma. Nature communications.

[37] Li Q, Li Z, Wei S, Wang W, Chen Z, Zhang L (2017). Overexpression of miR-584-5p inhibits proliferation and induces apoptosis by targeting WW domain-containing E3 ubiquitin protein ligase 1 in gastric cancer. Journal of experimental & clinical cancer research: CR.

