# Supplementary material for: Upregulation of SQLE Contributes to Poor Survival in Head and Neck Squamous Cell Carcinoma
Source: Int J Biol Sci. 2022 May 16;18(9):3576–91. doi: 10.7150/ijbs.68216 (PMC9254471; doi:10.7150/ijbs.68216)
Supplement: Supplementary file 1 — Supplementary figures. [file ijbsv18p3576s1.pdf]

Supplementary information

Upregulation of SQLE Contributes to Poor Survival in Head and Neck Squamous Cell Carcinoma

Supplementary Figures:

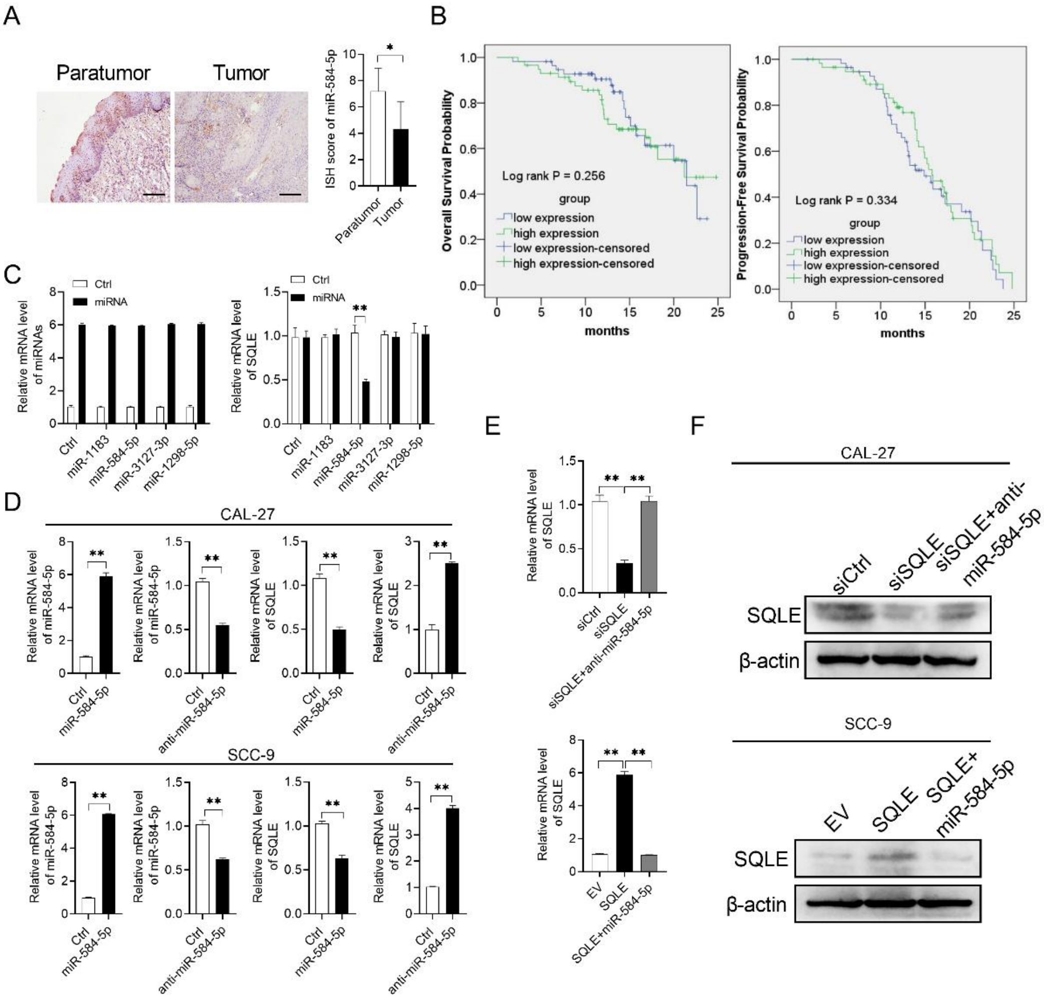

Figure S1. Expression levels of miR-584-5p in huamn HNSCC specimens and transfection effects of miR-584-5p in HNSCC cells.

(A) ISH analyses for miR-584-5p expression were performed in human HNSCC tissues. (B) Kaplan–Meier determination for OS and PFS according to miR-584-5p expression levels in our dataset. (C) qPCR analyses for candidate miRNAs transfection effects. (D) qPCR analyses for miR-584-5p transfection effects in HNSCC cells. (E-F) qPCR and WB analyses for SQLE expression levels of mRNA and protein in HNSCC cells treated as indicated.

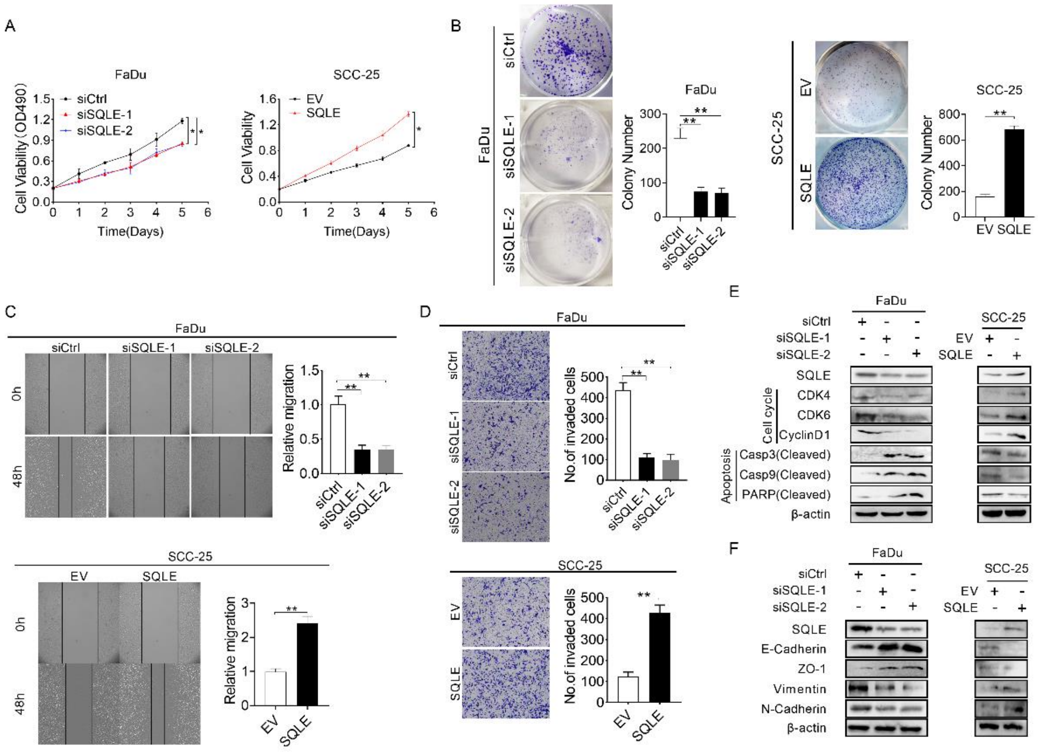

Figure S2. SQLE upregulation promoted HNSCC cells proliferation and metastasis *in vitro*.

(A-B) MTS and colony formation assays for determining the cell growth ability of HNSCC cell treated as indicated. (C-D) Wound healing and transwell invasion assays for evaluating the cell migration ability of FaDu and SCC-25 cells treated as specified. (E) Expression of markers related to cell proliferation and apoptosis detected in HNSCC cells by WB assessment. (F) WB analysis of EMT-related protein expression in HNSCC cells treated as indicated.

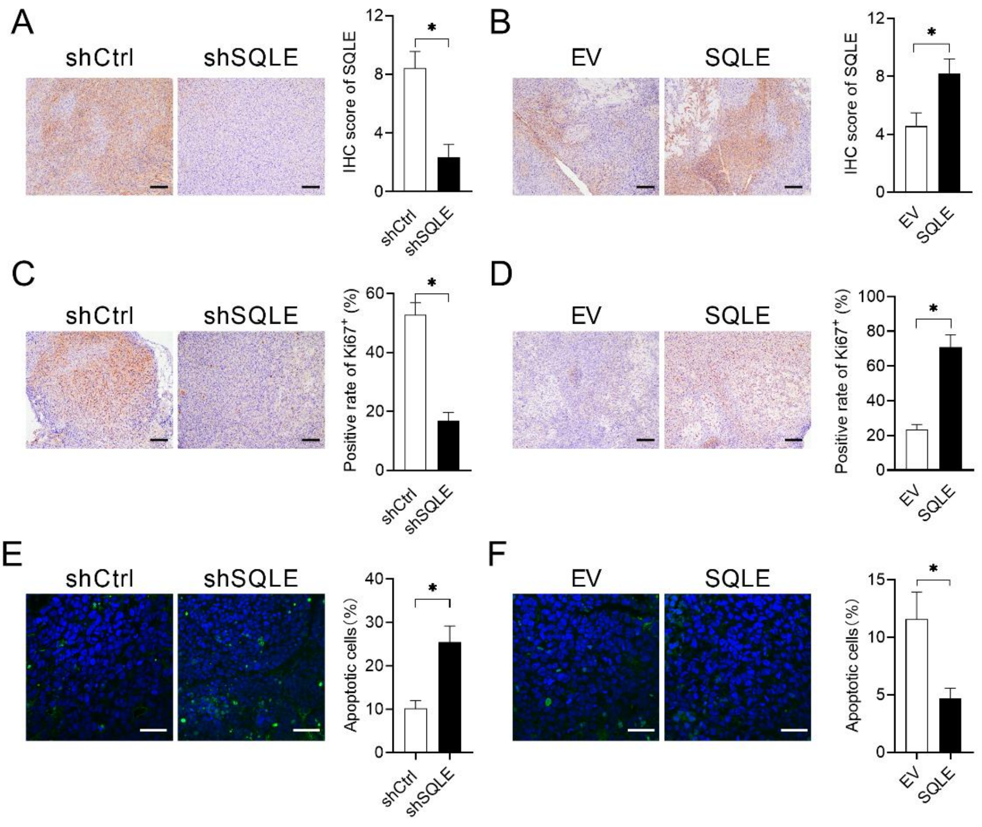

Figure S3. Morphologic examination for HNSCC cells xenograft tumors.
(A-B) IHC analyses for SQLE expression in xenograft tumors treated as indicated. (C-D) IHC analyses for Ki67 expression in xenograft tumors treated as indicated. (E-F) Tunel assays to analyze apoptotic cells rates in xenograft tumors treated as indicated.

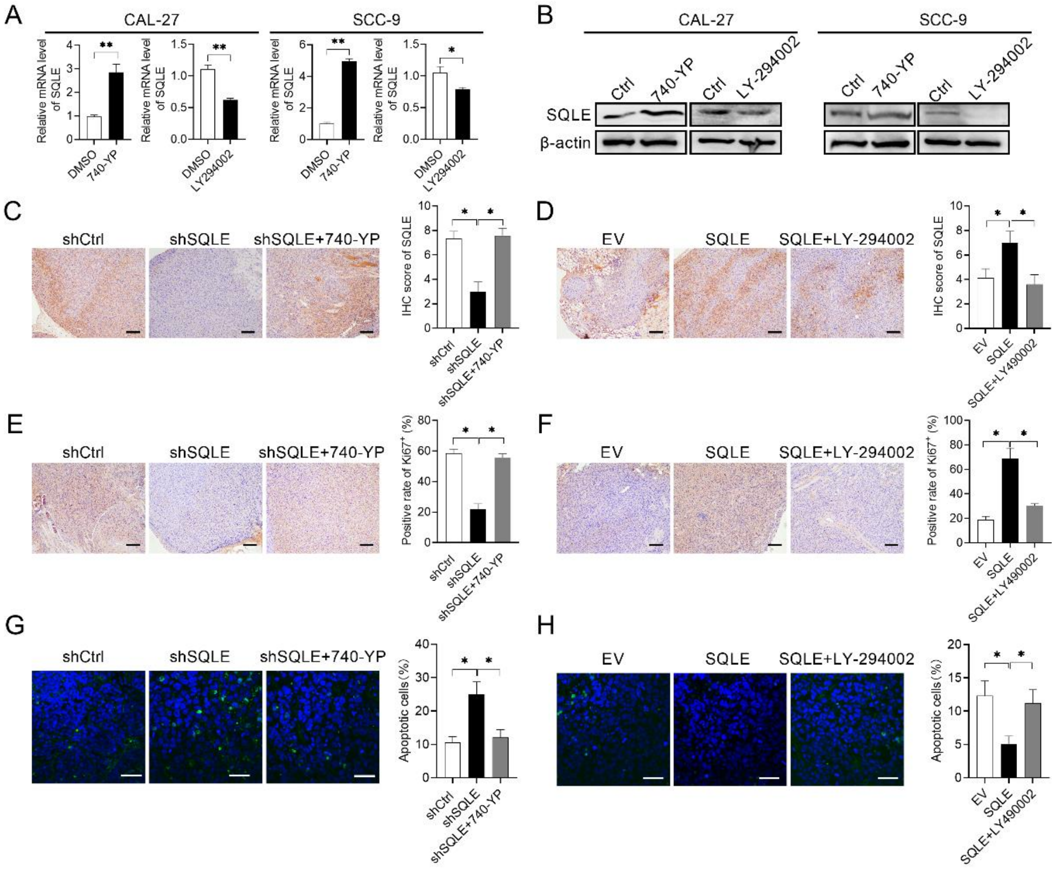

Figure S4. Effects of 740 Y-P and LY2940002 treatments on HNSCC cells *in vitro* and *in vivo*.

(A-B) qRT–PCR and western blotting analyses for SQLE expression were performed in HNSCC cells. (C-D) IHC analyses for SQLE expression in xenograft tumors treated as indicated. (E-F) IHC analyses for Ki67 expression in xenograft tumors treated as indicated. (G-H) TUNEL assays to analyze apoptotic cells rates in xenograft tumors treated as indicated.

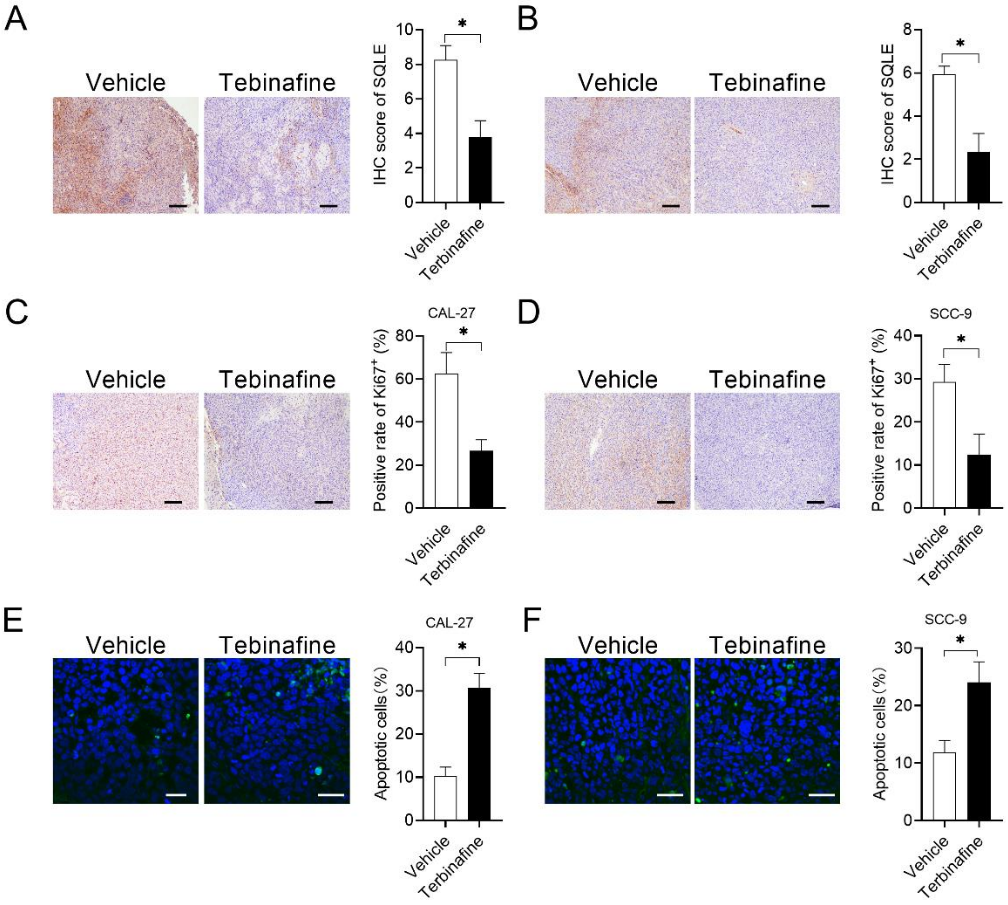

Figure S5. Morphologic examination for HNSCC cells xenograft tumors treated by terbinafine *in vivo*.

(A-B) IHC analyses for SQLE expression in xenograft tumors treated as indicated. (C-D) IHC analyses for Ki67 expression in xenograft tumors treated as indicated. (E-F) Tunel assays to analyze apoptotic cells rates in xenograft tumors treated as indicated.
